# Antagonistic drug interactions protect commensal *Bacteroidaceae* from macrolides via an RND-type efflux pump

**DOI:** 10.1080/19490976.2025.2596806

**Published:** 2025-12-09

**Authors:** Patrick Müller, Verena Schmidtchen, Jacobo de la Cuesta-Zuluaga, Lucía Pérez Jiménez, Cordula Gekeler, André Mateus, Lisa Maier

**Affiliations:** a Interfaculty Institute for Microbiology and Infection Medicine Tübingen, University of Tübingen, Tübingen, Germany; b Cluster of Excellence EXC 2124 Controlling Microbes to Fight Infections, University of Tübingen, Tübingen, Germany; c M3-Research Center for Malignome, Metabolome and Microbiome, University of Tübingen, Tübingen, Germany; d Department of Chemistry, Umeå University, Umeå, Sweden; e Department of Molecular Biology, Umeå University, Umeå, Sweden; f The Laboratory for Molecular Infection Medicine Sweden (MIMS), Umeå University, Umeå, Sweden

**Keywords:** *Bacteroidaceae*, non-antibiotic drugs, efflux pumps, antagonism

## Abstract

Antibiotics are essential to modern medicine, but their broad-spectrum activity can unintentionally disrupt the gut microbiota. This collateral damage may be alleviated by antagonistic drug interactions, in which specific compounds used in combination therapies selectively protect beneficial gut microbes from antibiotic activity. Using efflux pump inhibitors, transcriptomic and proteomic analyses, and targeted gene deletions, we show that a variety of non-antibiotic pharmaceuticals—from diverse therapeutic classes and at sub-inhibitory concentrations—can protect multiple *Bacteroidales* species from macrolide antibiotics. In *Bacteroidaceae*, this protection is mediated by a resistance-nodulation-division (RND)–type efflux pump, which is induced by the non-antibiotic drug but not by macrolides alone. Notably, protection persists even after the non-antibiotic drug is removed, and prolonged exposure results in stable macrolide resistance that is dependent on the RND-type efflux pump. Our findings illustrate how non-antibiotic drugs can inadvertently activate otherwise silent detoxification systems in gut microbes, uncovering resistance mechanisms that arise without antibiotic selection or gene transfer. While this can be harnessed to protect the microbiome during antibiotic therapy, it also reveals hidden resistance phenotypes that may escape detection in standard antimicrobial resistance assays.

## Introduction

Antibiotics have revolutionized modern medicine, but their broad-spectrum activity also disrupts the gut microbiota,^1^^,^^2^compromising microbiome functions^2‐10^ and driving the emergence and spread of antimicrobial resistance.^11^ Notably, similar off-target effects have been observed with a range of human-targeted drugs, including antidepressants, antipsychotics, proton pump inhibitors, and chemotherapeutics.^12^ These compounds can alter the composition and function of the gut microbiome, driving ecological shifts and the development of antimicrobial resistance. In addition, they create niches that enable the colonization of drug-resistant pathogens^13^ and promote recurrent infections.

To mitigate both the collateral damage and selective pressure that drugs exert on the gut microbiome, our previous work explored species-specific drug antagonism—a strategy in which a second compound selectively reduces a drug’s activity against commensals without impairing pathogen clearance.^14^ We focused on macrolides, widely prescribed antibiotics^15^ known to strongly impact gut commensals,^3‐5^ including *Bacteroidales.*^14^ Through compound screening, we identified three human-targeted drugs—dicumarol, benzbromarone, and tolfenamic acid—that selectively protected *Bacteroidales* from erythromycin *in vitro*, in synthetic and stool-derived communities, and in gnotobiotic mice, while retaining antibiotic efficacy against pathogens (and model organisms such as *Escherichia coli*). These protective effects were species-specific, shielding 42 to 65% of *Bacteroidales* and 41 to 65% *Bacteroides* OTUs in stool-derived communities, depending on the antagonist.^14^ Leveraging species-specific antagonism to protect gut commensal microbes during antibiotic treatment offers two main advantages: it protects in a manner dependent on the presence of the antagonist, and it lowers the selective pressure exerted by antibiotics. In particular, protecting *Bacteroidales* members, which are among the most prevalent and abundant gut commensal species, offers the potential advantages of retaining overall biomass and niche occupation in the gut microbiome. This makes it potentially harder for incoming pathogens (such as *Clostridioides difficile*) to colonize after antibiotic treatment.

However, the antagonists identified so far are themselves pharmaceutically active drugs, with dicumarol acting as an anticoagulant,^16^ benzbromarone as a uricosuric agent,^17^^,^^18^ and tolfenamic acid as a non-steroidal anti-inflammatory drug.^19^ For safe clinical application, antagonists must be optimized to minimize host effects and ensure their interactions with antibiotics are truly antagonistic—i.e., they must act temporarily and reversibly.

Here, we set out to investigate the mechanisms by which these antagonists confer protection to harness these pathways to the benefit of gut commensals. Using checkerboard growth assays, transcriptomics, proteomics, and targeted gene deletions, we show *in vitro* that *Bacteroidaceae* upregulate an RND-type efflux pump in response to the antagonists—but not in response to macrolides alone. These protective effects extended to azithromycin, a commonly used macrolide with a broader spectrum and longer half-life.^20^ Importantly, cells were not protected against the macrolide or the antagonist when tested individually, thereby masking its role in standard antimicrobial resistance assays. Notably, exposure to the antagonist induced lasting protection that persisted even after its removal and promoted the evolution of macrolide resistance.

Our findings indicate that these antagonists can temporarily protect *Bacteroidales* from macrolides, but they also reveal the risk that non-antibiotic drugs may unlock antibiotic resistances in gut microbes. This highlights the urgent need to evaluate all therapeutics—not just antibiotics—for their impact on the microbiome and resistance evolution.

## Materials and methods

### Bacterial cultivation

The species and strains used in this study are listed in Supplementary Table 1. They were purchased from DSMZ, generated in this study or a gift from the Typas lab (EMBL Heidelberg, Germany).

Bacterial cultivation was conducted as described before.^21^ In brief, all species and strains (except *E. coli* strains) were cultivated in mGAM medium (HyServe GmbH & Co.KG, Germany) at 37 °C. All media, glass, and plastic ware were pre-reduced for a minimum of 24 h under anaerobic conditions (2% H_2_, 12% CO_2_, 86% N_2_) in an anaerobic chamber (Coy Laboratory Products Inc.). *Escherichia coli* strains were cultivated in LB medium under aerobic conditions at 37 °C. Species were inoculated from frozen glycerol stocks into liquid culture medium and passaged twice (1:100) overnight before being used in subsequent experiments. To rule out possible contamination of species, their purity and identity were regularly checked via 16S rRNA sequencing and/or MALDI-TOF mass spectrometry (MS).^22^

Plasmid carrying *E. coli* BW23474 and *E. coli* DATC (Diaminopimelic acid-Auxotroph Transformation Conjugation)^23^ were incubated in LB medium with 100 µg/ml ampicillin and 0.3 mM diaminopimelic acid (DAP). For plasmid conjugation from *E. coli* DATC to *Phocaeicola vulgatus*, strains were streaked onto agar plates (LB with 100 µg/ml ampicillin and 0.3 mM DAP or mGAM respectively) and incubated at 37 °C overnight, under either aerobic conditions for *E. coli* or anaerobic for *P. vulgatus*. Conjugation was done on mGAM agar plates with 0.3 mM DAP under aerobic conditions at 37 °C for 18 h. Selection for plasmid-integrated *P. vulgatus* was done on mGAM agar plates with 50 µg/ml erythromycin and 200 µg/ml gentamicin anaerobically at 37 °C for 48 h. Counterselection was done on mGAM agar plates with 200 ng/ml anhydrotetracycline (aTc) for two subsequent 24 h incubations at 37 °C anaerobically.

### Compound sensitivity and antibiotic-compound interaction screening

Screening of compound sensitivities (here inhibitory concentration 90 (IC90)) and antibiotic-compound interactions, based on 8 × 8 checkerboards, was performed as described in our detailed protocol for screening compounds against gut bacteria^21^ and our previous studies.^14^^,^^24^

### Compound master plates

All compounds to be screened were diluted to 100x the desired assay concentration in dimethyl sulfoxide (DMSO) and transferred to a 96-well V-bottom plate (Greiner Bio-One, cat. no. 651261). For assessing inhibitory concentrations (IC), two-fold dilution series in DMSO were prepared from columns 1 to 11, with column 7 only containing DMSO as a control and column 12 serving as a blank. To assess compound interactions, we performed checkerboard analyzes. For those, a two-fold dilution series of the human-targeted drugs was conducted in DMSO from column 8 to 2, with columns 1, 9 and 12 only containing DMSO. Column 9 served as a control, and column 12 as a blank.

Compound master plates in 96-well V-bottom plates contained compounds in DMSO at 100x the assay concentrations and were sealed with aluminum foil seals for storage at −20 °C up to 3 months (Beckman Colter, cat. no. 538619).

### Compound screening plates

From the above-generated compound master plates, several assay screening plates were prepared. For this, a 96-deep-well plate was filled with mGAM medium, and the compound master plate was diluted 1:50 into it. Volumes were adjusted depending on the desired number of final screening plates. The compound-mGAM deep-well plates were aliquoted into 96-well U-bottom plates (Thermo Fisher Scientific, cat. no. Z168136) with 50 µl per well resulting in compound concentrations that were now 2x the desired assay concentration with 2% DMSO. Plates were sealed with aluminum foil seals and frozen at −20 °C until screening for a maximum of 3 weeks without thawing.

### Inhibitory concentration 90 (IC90) determination

Bacteria were grown in mGAM for two subsequent overnight cultures as described above. Plates were pre-reduced in the anaerobic chamber for 24 h before being inoculated with 50 µl of bacterial culture (OD 0.02) resulting in a final volume of 100 µl with 1x compound concentration, 1% DMSO and a bacterial starting OD of 0.01. Wells containing only 1% DMSO and bacteria served as growth controls. Plates were sealed with a breathable membrane (Sigma-Aldrich, cat. no. Z380059), and growth was measured every hour in a plate reader over 20 h. Simultaneous measurement of multiple plates was facilitated by using a microplate stacker coupled to the plate reader.

### Checkerboards for antagonistic antibiotic-compound interactions

Checkerboard screening plates containing 50 µl of 2x compound concentration and 2% DMSO were thawed one day before the screening. To screen compound interaction with antibiotics different 4x concentrations of the antibiotics were prepared in mGAM without DMSO. Twenty-five microliters of antibiotic dilutions were pipetted onto the checkerboard plates in column 1 to 8 with the lowest concentration in row G and the highest in row A. Row H and columns 9 to 12 were filled with 25 µl mGAM instead. The resulting checkerboard plates contained 75 µl with 1.33x compound concentration, 1.33x antibiotic concentration and 1.33% DMSO. Bacteria were grown in mGAM for two subsequent overnight cultures as described above. Plates were pre-reduced in the anaerobic chamber for 24 h before being inoculated with 25 µl bacterial culture (OD 0.04) in columns 1 to 9. Columns 10 to 12 were filled with 25 µl mGAM instead. Final volume was now 100 µl with 1x compound concentration, 1x antibiotic concentration, 1% DMSO and a bacterial starting OD of 0.01. Wells containing only 1% DMSO and bacteria served as growth controls. Plates were sealed with a breathable membrane (Sigma-Aldrich, cat. no. Z380059) and growth was measured every hour in a plate reader over 20 h. Simultaneous measurement of multiple plates was facilitated by using a microplate stacker coupled to the plate reader.

### Checkerboards with different salt concentrations or oxygen stress

Checkerboards to assess the effect of high salt or oxygen stress on antibiotic activity were performed as described above for antibiotic-compound interactions. To assess the effect of high salt, mGAM was enriched with 20% NaCl and a two-fold dilution series was conducted in mGAM from columns 8 to 2 before being aliquoted with 50 µl per well into 96-well plates. In the case of oxygen stress, 25 µl mGAM plates were prepared. Addition of the antibiotic dilutions was performed as described above, and plates were pre-reduced in the anaerobic chamber for 24 h.

In the case of high salt, mGAM plates were inoculated with 25 µl bacterial culture (OD 0.04) in columns 1 to 9. Columns 10 to 12 were filled with 25 µl mGAM instead. Final volume was now 100 µl with 1x salt concentration, 1x antibiotic concentration, 1% DMSO, and a bacterial starting OD of 0.01.

In the case of oxygen stress a two-fold dilution series of H_2_O_2_ was prepared inside the anaerobic chamber immediately before screening. Twenty-five microliters of those dilutions were then transferred to columns 2 to 8. Plates were inoculated with 25 µl of bacterial culture (OD 0.04) in columns 1 to 9. Columns 10 to 12 were filled with 50 µl mGAM instead. The final volume was now 100 µl with 1x H_2_O_2_ concentration, 1x antibiotic concentration, 1% DMSO and a bacterial starting OD of 0.01.

Wells containing only 1% DMSO and bacteria served as growth controls. Plates were sealed with a breathable membrane (Sigma-Aldrich, cat. no. Z380059) and growth was measured every hour in a plate reader over 20 h. Simultaneous measurement of multiple plates was facilitated by using a microplate stacker coupled to the plate reader.

### Checkerboards with efflux-pump inhibitors

Checkerboards investigating the effect of the efflux-pump inhibitors reserpine, verapamil, and carbonyl cyanide-m-chlorophenylhydrazone (CCCP) were prepared as described above, with the difference that the human-targeted drugs were diluted 1:25 in the deep-well plates, and 25 µl of human-targeted drug dilutions were added to the plates. Preparation of the antibiotic dilutions was the same. Additionally, one day before the screening, together with the antibiotics, efflux-pump inhibitors were diluted to 4x their desired assay concentration (desired conc.: 20 µg/ml for reserpine and verapamil, 100 µM for CCCP) in mGAM, and 25 µl were added to columns 1 to 8 of the plates. Plates were pre-reduced in the anaerobic chamber for 24 h before being inoculated with 25 µl of bacterial culture (OD 0.04) in columns 1 to 9. Columns 10 to 12 were filled with 25 µl mGAM instead. Final volume was now 100 µl with 1x compound concentration, 1x antibiotic concentration, 1x efflux-pump inhibitor concentration, 1% DMSO, and a bacterial starting OD of 0.01. Wells containing only 1% DMSO and bacteria served as growth controls. Plates were sealed with a breathable membrane (Sigma-Aldrich, cat. no. Z380059), and growth was measured every hour in a plate reader over 20 h. Simultaneous measurement of multiple plates was facilitated by using a stacker coupled to the plate reader.

### Growth curve analysis

Analysis of growth curves was done using the *R* package ‘neckaR’ (https://github.com/Lisa-Maier-Lab/neckaR) as described before.^14^^,^^21^^,^^24^ Growth was analyzed by calculating the area under the curves (AUCs) of all growth curves and normalizing them to the control wells containing only bacteria and DMSO (in case of compounds). The median AUC was calculated for each condition across all replicates. In case of compound sensitivity screening IC90 was defined as the lowest concentration at which a median AUC < 0.1 was observed.

### Bliss independence interaction scores

For the checkerboard analyzes, in addition to the AUCs, Bliss independence interaction scores (also called “Excess over Bliss scores'') were calculated. Bliss scores indicate whether two drugs act synergistically, antagonistically, or independently from one another, assuming that mostly the fitness ( = growth) in the presence of one drug equals that in the presence of both drugs divided by the fitness in the presence of the other drug alone.^25^^,^^26^ The Bliss score calculation is based on *Brochado et al.* ^26^ and *Liu et al.* ^27^ and uses the above calculated AUCs (as an indicator for fitness) to determine antagonism, synergy or independence. Bliss scores are analyzed by plate, first determining the predicted fitness of compound-compound combinations. For this, the observed fitness of compound A at concentration a (AUC_Aa_) is multiplied by the observed fitness of compound B at concentration b (AUC_Bb_):

(1) $$\mathrm{Predicted Fitness}(F_{\mathrm{AaBb}})\;=\mathrm{AU}C_{\mathrm{Aa}}\;*\;\mathrm{AU}C_{\mathrm{Bb}}$$

To calculate the Excess over Bliss score, the predicted fitness is subtracted from the observed fitness, which is the AUC of the combination of compound A at concentration a and compound B at concentration b (AUC_AaBb_):

(2) $$\mathrm{Excess over Bliss score}(I)\;=\mathrm{AU}C_{\mathrm{AaBb}}\;-\;F_{\mathrm{AaBb}}$$

An Excess over Bliss score I>0 denotes antagonism, I<0 synergy, and I=0 independence of both compounds.

To focus on the relevant combinations only conditions one concentration step below the antibiotics IC90 and up to two steps above the IC90 were plotted. Whether a condition denotes independence or not was determined by setting ±1.5-times interquartile ratio (IQR) of the distribution of all Bliss scores per antibiotic as the range of Bliss independence. Conditions were further defined as “Protection” if their Excess over Bliss score was >1.5-times IQR of all Bliss scores and had a predicted fitness (F_AaBb_) <0.1.

### Tanimoto scores of chemical similarity

To measure chemical similarities of compounds, several similarity measures are available. We used the Tanimoto score, which assesses chemical similarity based on binary 2D-molecular fingerprints. For this, we used the *R* package ‘RCDK’, which is an *R* implementation of the widely used chemoinformatics ‘CDK’ library for Java. Simplified molecular-input line-entry system (SMILES) of each molecule was taken from PubChem (https://pubchem.ncbi.nlm.nih.gov/) to generate molecular fingerprints using default parameters (type = “standard”). With these fingerprints, similarity matrices were generated using the default parameters to retrieve the Tanimoto scores. Tanimoto scores range from 0 (no similarity) to 1 (identical molecules).

### Expression analyzes after antibiotic and/or human-targeted drug treatment

For gene and protein expression analysis after drug treatment, bacteria were grown in mGAM for two subsequent overnight cultures as described above. They were subsequently diluted 1:100 into 50 ml mGAM and incubated at 37 °C anaerobically until mid-exponential phase. Culture OD was measured to ensure mid-exponential phase, and one culture flask corresponding to one strain and one replicate was aliquoted into 4 × 10 ml samples. To the 10 ml samples, 10 µl of either dicumarol, erythromycin, or both drugs were added to a final concentration of 10 µM (dicumarol) and 0.65 µM (erythromycin). For proteomics analyzes, benzbromarone at 10 µM and tolfenamic acid at 20 µM were also investigated. One sample was left untreated as a control. All samples were incubated for an additional 30 min at 37 °C anaerobically before being transferred from the anaerobic chamber onto ice.

Samples were centrifuged at 4 °C for 5 min at 4,000g, and 8 ml of the supernatant was discarded. The pellet was resuspended in the remaining volume (~2 ml) on ice and aliquoted 2x into 1 ml tubes. The duplicate samples were centrifuged at 4 °C for 5 min at 4,000g and the supernatant was completely removed.

For RNA-Seq analysis, cell pellets were snap-frozen in liquid nitrogen and stored at −80 °C until RNA extraction. For proteomics analyzes, cell pellets were resuspended in 500 µl of 2% sodium dodecyl sulfate (SDS) and boiled at 95 °C for 15 to 20 min before being stored at −20 °C for downstream analysis.

### Genome annotations

For analyzes of the strain *P. vulgatus* ATCC 8482, we used the reference genome assembly, genome annotation and predicted transcriptome available in the Ensembl! bacteria database (GenBank ID: GCA_000012825.1).

### RNA-Seq analysis

RNA extraction, rRNA depletion, library preparation and RNA sequencing was performed by the NGS Competence Center Tübingen (NCCT) using standard protocols and NextSeq 500 Mid Output Kit v2.5 (150 cycles).

RNA-seq analysis was conducted using the nf-core rna-seq v.3.11.1 pipeline^28^ and nextflow v.22.10.6.4. In brief, adapters were removed from the raw sequencing reads using Trim Galore v.0.6.7,^29^ and quality reports for each sample were created with fastqc v.0.11.9 (https://github.com/s-andrews/FastQC) and multiQC v.1.14.^30^ To quantify the expression of transcripts, a pseudo alignment against the reference genome and transcriptome was performed using Salmon v.1.10.1.^31^ Tables of raw transcript counts were used for downstream analyzes.

Differential expression analysis was conducted with the *R* package ‘DESeq2’ using the standard workflow.^32^ Gene expression of treated samples was compared to the untreated controls within one strain and one replicate. Shrinkage of effect size (LFC estimates) for log2 fold changes was performed using *apeglm*^33^ to remove the noise associated with log2 fold changes from low count genes without requiring arbitrary filtering thresholds, and s-values were calculated. Genes were considered a hit if |log2 fold change| > 1 and s-value < 0.005.

### Proteomics analysis

Lysates (see *Expression analyzes after antibiotic and/or human-targeted drug treatment*) were digested with a modified sp3 protocol,^34^ as previously described.^35^ Briefly, samples were added to a bead suspension (10 μg of beads (Sera-Mag Speed Beads, 4515−2105−050250, 6515−2105−050250) in 10 μl of 15% formic acid and 30 μl ethanol) and incubated shaking for 15 min at room temperature. Beads were then washed four times with 70% ethanol. Proteins were digested overnight by adding 40 μl of 5 mM chloroacetamide, 1.25 mM TCEP, and 200 ng trypsin in 100 mM HEPES pH 8.5. Peptides were eluted from the beads and dried under a vacuum. Peptides were then labeled with TMTpro (Thermo Fisher Scientific), pooled and desalted with solid-phase extraction using a Waters OASIS HLB Elution Plate (30 μm). Samples were fractionated onto 48 fractions on a reversed-phase C18 system running under high pH conditions, pooling every sixth fraction together. Samples were analyzed by LC-MS/MS using a data dependent acquisition strategy on a Thermo Fisher Scientific Vanquish Neo LC coupled with a Thermo Fisher Scientific Orbitrap Exploris 480. Raw files were processed with MSFragger against the *P. vulgatus* FASTA database downloaded from UniProt (UP000002861) using standard settings for TMT. Protein levels of treated samples were compared to untreated controls and log2 fold changes as well as *p*-values were calculated using limma.^36^ Proteins were considered a hit if |log2 fold change| > 0.585 and adjusted *p*-value < 0.05.

### Generating genomic knockouts in *P*. *vulgatus* and *B. uniformis*

Genomic knockouts of target genes in *Phocaeicola vulgatus* or *Bacteroides uniformis* were generated based on the method described in García-Bayona et al., 2019.^37^ This method relies on a two-step allelic exchange by homologous recombination. For this, the regions flanking the gene of interest ~1,500 bp (*P. vulgatus*) or ~1,000 bp (*B. uniformis*) up- and downstream were amplified from genomic DNA and cloned into the linearized anhydrotetracycline (aTc)-inducible suicide vector pLGB13^37^ using HiFi/Gibson Assembly. This plasmid contains an ampicillin resistance for maintenance in *E. coli*, an erythromycin resistance cassette as a selection marker in *Bacteroidetes* and the aTc-inducible ssBfe1 counter selection cassette which will express the highly toxic effector Bfe1 from the Type VI secretion system of *Bacteroides fragilis.*^38^ The vector containing the flanking regions was transformed into *E. coli* DATC^23^ using heat shock transformation. Upon successful transformation the plasmid was conjugated into *P. vulgatus* where it integrates into the chromosome at the site of the gene of interest via homologous recombination under erythromycin selection. Using aTc-counter selection, which induces ssBfe1-mediated rapid cell death, colonies were selected that underwent a second homologous recombination, resulting in the loss of the integrated plasmid again. Colonies were analyzed via PCR and Sanger sequencing to check whether they were wild-type revertants or knockouts. Primer and plasmids can be found in Supplementary Tables 6 and 7.

### Dicumarol induction experiment

*P. vulgatus* strains (type strain and RND-type pump knockout) were induced with 20 µM dicumarol or 1% DMSO as a control. The OD of overnight cultures of the strains was measured and they were subcultured at OD 0.02 into fresh media with either 20 µM Dicumarol or 1% DMSO for 30 min, 1 h and 2 h at 37 °C. Cells were centrifuged at 1,000 g for 4 min and washed with 1 ml PBS before being transferred to a 96-well plate containing different concentrations of erythromycin or azithromycin (see also *Inhibitory concentration 90 (IC90) determination* above). Plates were sealed with a breathable membrane (Sigma-Aldrich, cat. no. Z380059) and growth was measured every hour in a plate reader over 20 h. Simultaneous measurement of multiple plates was facilitated by using a stacker coupled to the plate reader.

### Dicumarol evolution experiment

*P. vulgatus* strains (type strain and RND-type pump knockout) were evolved with dicumarol or DMSO as a control. Overnight cultures of the strains were diluted 1:600 into deep well plates containing mGAM with 0.25x IC90 of dicumarol (20 µM) or **1%** DMSO. Every 24 h, these cultures were subcultured 1:600 into deep well plates with mGAM containing increasing amounts of dicumarol or **1%** DMSO. Dicumarol concentrations were increased daily by 2-fold all the way to 16x IC90 (1,280 µM). For each strain, three independent lineages were evolved. Each day the evolved lines were stored as cryo stocks for downstream use.

### Dose-response curves of dicumarol-induced or dicumarol-evolved lineages

To assess whether induction with dicumarol or evolution to it increased resistance to the macrolides, growth curves were measured. The background was subtracted and the maximum OD determined. For the dicumarol-induction experiment, growth curves were mostly smooth, and the maximum OD could simply be calculated. For the dicumarol-evolved lines, growth curves in parts had substantial spikes which is why the calculation of the maximum OD had to be adapted. In this case the highest OD value of the growth curve was removed to account for some OD spikes and from the remaining top five values, the median was calculated, which resulted in the maximum OD for each well. For both experiments, maximum OD values were normalized to the median of the untreated control wells per plate. Dose-response curves with corresponding IC50 values were generated using the *drc* package^39^ in R.

## Results

### Certain human-targeted drugs protect various *Bacteroidales* species from the macrolides erythromycin and azithromycin

**Figure 1.**
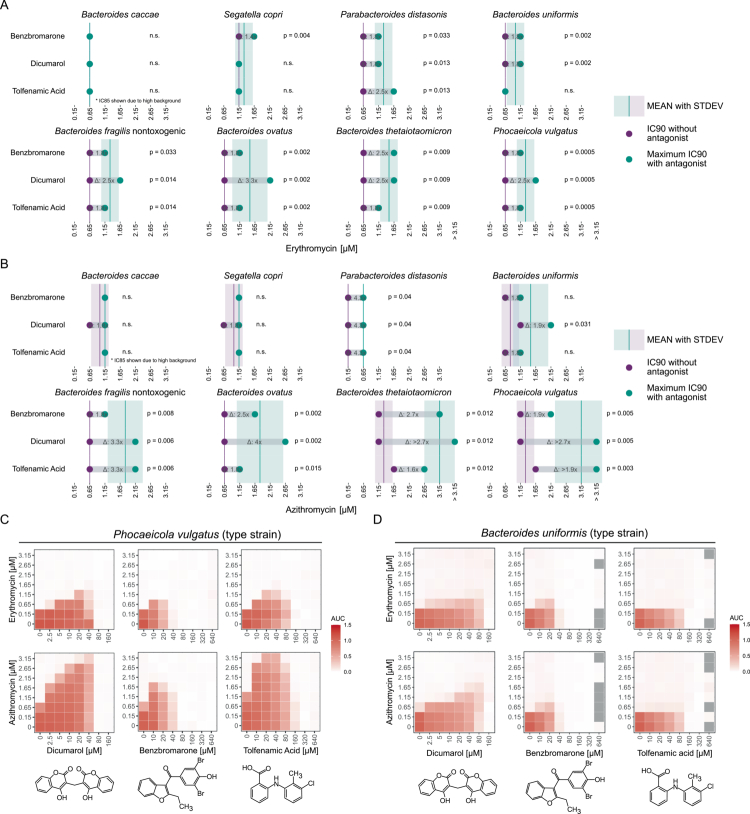
Antagonists protect against the macrolides erythromycin and azithromycin across a variety of *Bacteroidales* species. **A-B)** Shift in inhibitory concentration (IC) 90 of **A)** erythromycin or **B)** azithromycin against eight *Bacteroidales* upon exposure to the three human-targeted drugs dicumarol, benzbromarone or tolfenamic acid. Shown is for each strain the IC90 of three biological replicates of the macrolide in mGAM in the absence (purple dots) or presence (green dots) of the three different human-targeted drugs. Per strain, the mean IC90 and standard deviation of the macrolide with (green line and box) or without (purple line and box) human-targeted drugs is given across all three drugs. For human-targeted drugs that cause a change in IC90, the fold change in IC90 is given as a gray bar. Note that for *B. caccae* IC85 had to be calculated (see Supplementary Figure 1). Significant differences in IC90 were assessed using the Wilcoxon rank-sum test. *P*-values were adjusted for multiple comparisons using the Benjamini-Hochberg (FDR) method. **C-D)** Checkerboard analyzes of **C)** *P. vulgatus* type strain or **D)** *B. uniformis* type strain grown in mGAM with increasing concentrations of the macrolide erythromycin or azithromycin in combination with increasing concentrations of the three human-targeted drugs dicumarol, benzbromarone, and tolfenamic acid. Heatmaps show the median area under the growth curve (AUC) of three biological replicates normalized to the growth of the unperturbed control. Gray tiles indicate missing conditions that failed quality control.

We previously identified three human-targeted drugs that impaired the activity of the macrolide erythromycin against *Bacteroidales*: dicumarol, benzbromarone, and tolfenamic acid.^14^ The extent of this protection varied depending on the species, with *Phocaeicola vulgatus* (formerly *Bacteroides vulgatus*) and *Bacteroides thetaiotaomicron* showing a strong reduction in erythromycin efficacy upon treatment with these antagonists, and species such as *Bacteroides caccae* exhibiting no change in susceptibility to erythromycin under the same conditions (Figure 1A).

To assess whether protection from erythromycin also extends to other macrolides, we examined the effects of these antagonists on azithromycin, a clinically highly relevant macrolide, in eight *Bacteroidales* species: *Phocaeicola vulgatus*, *Bacteroides uniformis*, *Bacteroides fragilis* (nontoxigenic), *Bacteroides thetaiotaomicron*, *Segatella copri* (formerly *Prevotella copri*), *Bacteroides caccae*, *Bacteroides ovatus*, and *Parabacteroides distasonis* (Supplementary Table 1). To this end, we performed checkerboard analyzes, evaluating bacterial growth (quantified by area under the curve (AUC); see Methods) in response to pairwise combinations of azithromycin with each antagonist across multiple concentrations. All three compounds enabled bacterial growth at higher azithromycin concentrations in several species—including *P. vulgatus*, *B. fragilis*, *B. uniformis*, *B. thetaiotaomicron*, and *B. ovatus*—thereby attenuating the antibiotic’s inhibitory effect (Figure 1B–D & Supplementary Figure 1). The protective effects of the antagonists against azithromycin manifested at concentrations previously shown to protect against erythromycin^14^ (Figure 1B–D). Protection from azithromycin extended across multiple concentration steps beyond the IC90 (i.e., 90% growth inhibition), allowing growth at higher antibiotic levels—for example with dicumarol, *P. vulgatus* grew at >3.15 µM azithromycin (at least 2.7-times increase in IC90) compared to a maximum of 1.15 µM erythromycin (2.5-times increase in IC90) (Figure 1A & B). As with erythromycin, protection was species-specific: *S. copri* and *B. caccae* showed no significant increase in azithromycin IC90, *P. distasonis* showed only minimal, yet significant, protection (Figure 1B and Supplementary Figure 1), whereas *P. vulgatus* and *B. thetaiotaomicron* exhibited multi-step increases in IC90. These two species sustained growth even at the highest tested azithromycin concentration in the presence of dicumarol or tolfenamic acid (Figure 1B & C). These findings align with our previous observations for erythromycin, where the extent of protection varied and *B. caccae* remained unresponsive^14^ (Figure 1A). Overall, antagonists mediated similar protection against both erythromycin and azithromycin.

To investigate whether structural analogs of the antagonists could also confer macrolide protection, we tested warfarin—a 4-hydroxycoumarin anticoagulant structurally related to dicumarol. Warfarin provided weaker protection against azithromycin: in *P. vulgatus*, it modestly increased the azithromycin IC90, but had no effect on erythromycin susceptibility (Supplementary Figure 2A). Only *B. thetaiotaomicron* showed a >2-step increase in azithromycin IC90, while most other species exhibited minimal or no response (Supplementary Figure 2B). To explore this further, we screened 16 additional compounds, including structurally similar coumarin analogs, super-warfarins, and the related indandione pindone, for erythromycin protection in *P. vulgatus* (Supplementary Figure 3A). None of the compounds increased erythromycin IC90 (Supplementary Figure 3B), indicating that the coumarin moiety alone is insufficient to confer macrolide protection. Therefore, structural similarity alone is not a reliable predictor for identifying human-targeted drugs that protect *P. vulgatus* from macrolides.

Since structurally diverse compounds could induce macrolide protection while structurally similar ones could not, we hypothesized that protection might arise from a global cellular stress response rather than a specific pathway. To test this, we exposed *P. vulgatus* to high salt (NaCl) and oxidative stress (H₂O₂), but neither condition conferred protection against erythromycin (Supplementary Figure 4).

These results indicate that human-targeted drugs from pharmacologically and chemically diverse classes can protect *Bacteroidales* from macrolides, with the extent of protection varying by compound and species.

### Antagonists, but not macrolides, upregulate an RND-type efflux pump in *Bacteroidaceae*

**Figure 2.**
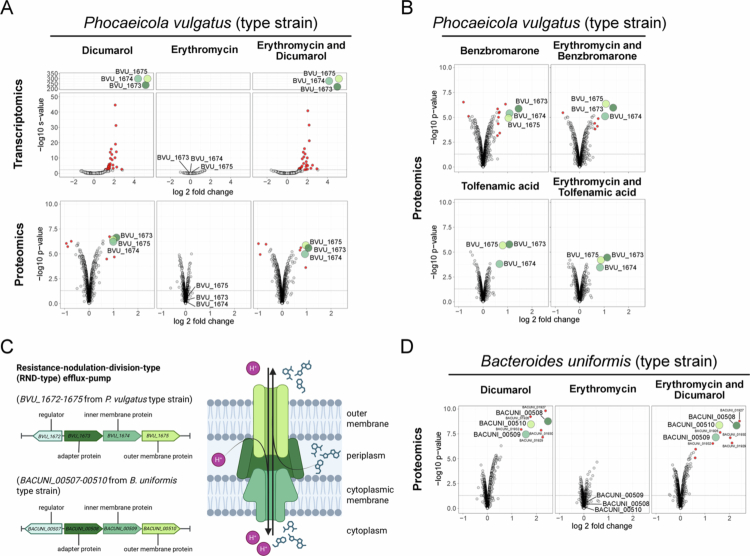
*P. vulgatus* and *B. uniformis* upregulate an RND-type efflux pump in response to the antagonists but not the macrolide. *P. vulgatus* and *B. uniformis* type strains were grown to mid-exponential phase in mGAM and treated for 30 minutes with the human-targeted drugs, the macrolide erythromycin, or a combination of both in three biological replicates. **A)** RNA-Seq analysis (top) or proteomics analysis (bottom) of *P. vulgatus* type strain treated with either dicumarol (10 µM), erythromycin (0.65 µM) or both drugs. Volcano plots show the log2 fold change in gene expression or protein abundance compared to untreated controls on the x-axis. Significance levels, as denoted by s-values for transcriptomics and *p*-values for proteomics, are shown on the y-axis log10 transformed. Colored dots indicate significantly altered gene or protein levels. For transcriptomics significance was set to |log2 fold change| >1 and s-value < 0.005. For proteomics significance was set to |log2 fold change| >0.585 and adjusted *p*-value <0.05. Dotted lines indicate significant s- or *p*-value cutoffs. **B)** Proteomics analysis of *P. vulgatus* type strain treated with either benzbromarone (10 µM), tolfenamic acid (20 µM), erythromycin (0.65 µM) or a combination of drugs. **C)** Schematic illustration of the operon structure and protein complex of the top hit (BVU_1673−1675) in *P. vulgatus* and (BACUNI_00508−00510) in *B. uniformis* when treated with the human-targeted drugs. The individual proteins of this RND-type efflux pump are always highlighted in **A), B),** and **D)** with the same color. Created with BioRender (https://BioRender.com/uwudgbr). D) Proteomics analysis of *B. uniformis* type strain treated with either dicumarol (10 µM), erythromycin (0.65 µM) or both drugs. Additionally, the ABC-type BACUNI_01926−1932 which is upregulated in the presence of dicumarol is indicated.

To examine the response of *Bacteroidaceae* to antagonists, we performed transcriptomic and proteomic analyzes of *P. vulgatus*. Mid-exponential phase cells were treated for 30 minutes with dicumarol (10 µM), benzbromarone (10 µM), tolfenamic acid (20 µM), erythromycin (0.65 µM), or combinations of each human-targeted drug with erythromycin. These concentrations were chosen based on their ability to induce protection from macrolides **(**Figure 1C). Dicumarol significantly altered the expression of 29 genes, while erythromycin alone had no significant effect; their combination affected 30 genes (Figure 2A**,** Supplementary Table 2). At the protein level, dicumarol and its combination with erythromycin altered the abundance of 9 proteins, while erythromycin alone had no significant impact (Figure 2A, Supplementary Table 3). Benzbromarone and tolfenamic acid changed 13 and 3 proteins, respectively, with similar patterns observed when combined with erythromycin (8 and 3 proteins; Figure 2B, Supplementary Table 3).

Among the genes identified by transcriptomic and proteomic analyzes, one operon (*BVU_1673–1675*) showed notably strong upregulation in response to the human-targeted drugs. Dicumarol triggered the highest induction, with up to a 39.88-fold increase in transcript levels and a 2.17-fold increase in protein abundance. Benzbromarone and tolfenamic acid also elevated protein levels by 2.73- and 2.19-fold, respectively (Figure 2A & B). In contrast, erythromycin alone did not affect the expression of this operon. The *BVU_1673–1675* operon encodes a resistance-nodulation-division (RND)-type efflux pump in *P. vulgatus* (Figure 2C), a tripartite system consisting of an inner membrane transporter (BVU_1674), a periplasmic adapter protein (BVU_1673), and an outer membrane channel (BVU_1675). These pumps utilize the proton motive force to export compounds, such as drugs, from the cytosol or periplasm to the extracellular environment.^40^

To corroborate these findings in a species other than *P. vulgatus*, we examined protein-level changes in *B. uniformis*. Despite the fact that *B. uniformis* is less responsive to the human-targeted drugs and mainly dicumarol is able to protect it from macrolides (Figure 1A, B & D), this species is after *P. vulgatus* one of the most prevalent and abundant gut commensal. Consistent with results from *P. vulgatus*, *B. uniformis* showed significant upregulation of proteins encoded by the *BACUNI_00508–00510* operon, with a fold change of 5.58 in response to dicumarol but not to erythromycin alone (Figure 2D, Supplementary Table 4). This operon encodes the homologous RND-type efflux pump to *BVU_1673–1675* in *P. vulgatus*: *BACUNI_00509* encodes the inner membrane transporter, *BACUNI_00508* the adapter protein, and *BACUNI_00510* the outer membrane channel.

These findings suggest that upregulation of an RND-type efflux pump in *Bacteroidaceae* may increase macrolide export, thereby reducing susceptibility to macrolide antibiotics.

### The RND-type efflux pump BVU_1673−1675 is essential for human-targeted drug–mediated protection from macrolides

**Figure 3.**
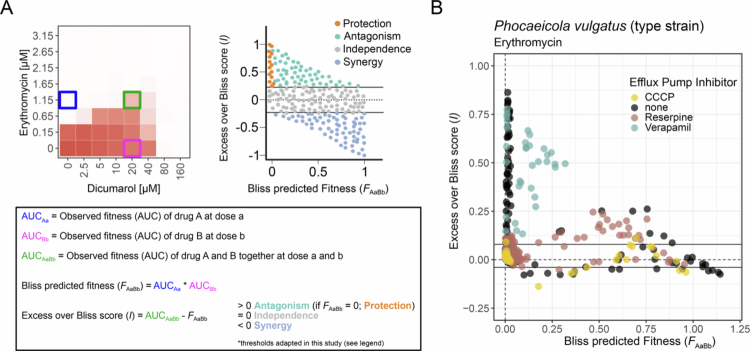
Efflux-pump inhibitors block protection from macrolides by antagonists. **A)** Schematic overview of the Bliss Score analysis (see also methods). Excess over Bliss scores indicate whether two drugs act synergistically, antagonistically, or independently from one another. They are calculated by subtracting the predicted fitness of a drug combination (*F*_AaBb_) from the observed fitness (AUC_AaBb_). The predicted fitness of a combination is defined as the product of the individual fitness with each drug alone (AUC_Aa_ x AUC_Bb_). Bliss scores >0 indicate antagonism, = 0 independence, and <0 synergy. Importantly, protection (a form of antagonism) occurs at Bliss scores >0 that have a predicted fitness of 0. In this study, thresholds were adapted as optical densities are never exactly 0. We used the 1.5-times interquartile ratio (IQR) of the distribution of all Bliss scores as the range of Bliss independence, meaning that Bliss scores >1.5x IQR denote antagonism. Furthermore, for protection, the threshold for the predicted fitness was set to be below 0.1 instead of exactly 0. Top right panel created with BioRender.com. **B)** Bliss scores of *P. vulgatus* type strain treated with erythromycin in combination with the three human-targeted drugs dicumarol, benzbromarone or tolfenamic acid, with the addition of different efflux pump inhibitors in three biological replicates: none (black), 20 µg/mL reserpine (brownish-pink), 20 µg/mL verapamil (green turquoise) or 100 µM carbonyl cyanide-m-chlorophenylhydrazone (CCCP) (yellow). CCCP was only tested with erythromycin-dicumarol. The x-axis shows the predicted fitness of a condition, while the y-axis shows the Excess over Bliss Score. Horizontal lines indicate the ±1.5-times IQR of the distribution of all Bliss scores as the range of Bliss independence.

Given the upregulation of an RND-type efflux pump by antagonistic human-targeted drugs, we investigated the contribution of efflux pumps to the drug-mediated protection from macrolides. Checkerboard assays with erythromycin and the three antagonists were performed in *P. vulgatus*, with the addition of verapamil (20 µg/mL), reserpine (20 µg/mL), or CCCP (100 µM; tested only with dicumarol). Verapamil and reserpine inhibit efflux pumps by direct binding, while CCCP disrupts the proton gradient as a protonophore. Using Excess over Bliss scores—a metric assessing drug interaction types (protection and changes in antibiotic susceptibility—either decreased (antagonism) or increased (synergy), Figure 3A; Methods)—we evaluated changes in macrolide-antagonist interactions upon efflux inhibition. Analyzes focused on erythromycin concentrations from one step below to two steps above its IC90 (0.15 µM to 1.65 µM).

Without efflux pump inhibitors (Figure 3B, black points), 48 of 252 (19%) conditions resulted in cross-protection, and additional 8 (3.2%) conditions showed reduced erythromycin susceptibility (antagonism). Verapamil treatment (Figure 3B, turquoise points) lowered the amount of protective conditions to 15 of 216 (6.9%) and shifted many to ‘independence’ or ‘antagonism’, meaning a slight increase in AUC without changes in IC90 (18 of 216, 8.3%). Reserpine showed a similar effect (Figure 3B, brownish-pink points), reducing protection to 12 of 216 (5.6%) conditions and causing reduced susceptibility/antagonism in 22 (10.2%). For CCCP, tested only with dicumarol (Figure 3B, yellow points), protection almost disappeared (1 of 72, 1.4%), reduced susceptibility/antagonism was minimal (1 of 72, 1.4%), and most conditions (65 of 72, 90.3%) showed no interaction. In comparison, dicumarol alone (no inhibitor) led to protection in 23 of 84 (27.4%) and reduced susceptibility/antagonism in 4 (4.8%) conditions. In summary, the presence of efflux pump inhibitors substantially reduces the cross-protection conferred by human-targeted drugs against macrolides.

**Figure 4.**
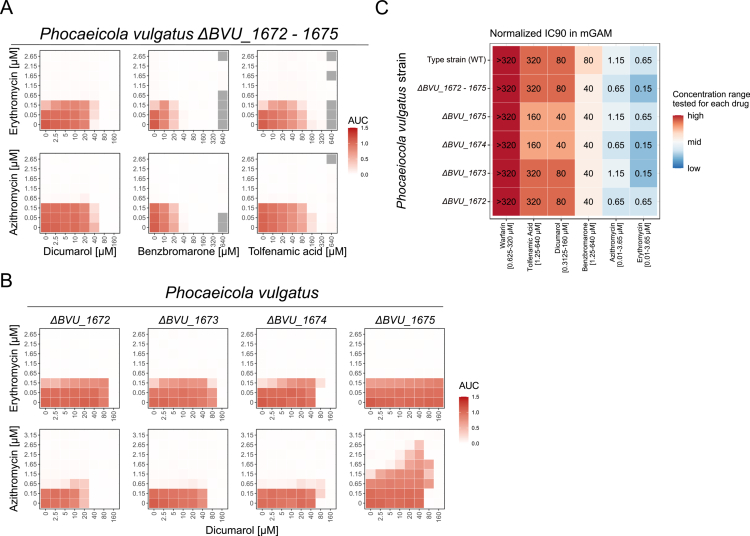
The RND-type efflux pump BVU_1673−1675 in *P. vulgatus* is essential for antagonist-mediated protection from macrolides. **A-B)** Checkerboard analysis of *P. vulgatus* grown in mGAM with increasing concentrations of the macrolide erythromycin or azithromycin in combination with increasing concentrations of dicumarol, benzbromarone or tolfenamic acid. Heatmaps show the median area under the growth curve (AUC) of three biological replicates normalized to unperturbed control. Gray tiles indicate missing conditions that failed quality control. **A)** Whole operon *BVU_1672−1675* knockout, **B)** single gene knockouts of the operon *BVU_1672−1675*. **C)** Inhibitory concentration (IC) 90 values of the human-targeted drugs and the two macrolides erythromycin and azithromycin on *P. vulgatus* type strain and knockout strains in mGAM. IC90 values are calculated based on the median normalized AUC of three biological replicates, with an AUC < 0.1 indicating the IC90. Heatmap shows the IC90 values in µM with a color code corresponding to the concentration range tested per drug. The label “>” means that even the highest concentration tested did not inhibit the strain by 90% or more (no IC90 reached).

**Figure 5.**
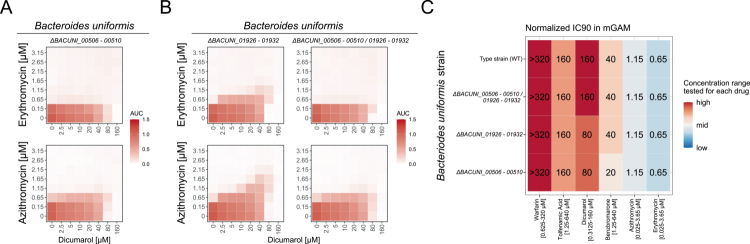
In *B. uniformis*, a homolog of the *P. vulgatus* RND-type efflux pump is essential for macrolide protection by antagonists. **A-B)** Checkerboard analyzes of *B. uniformis* operon knockout strains grown in mGAM with increasing concentrations of the macrolide erythromycin or azithromycin in combination with increasing concentrations of dicumarol, benzbromarone or tolfenamic acid. Heatmaps show the median area under the growth curve (AUC) of three biological replicates normalized to the growth of the unperturbed control. Gray tiles indicate missing conditions that failed quality control. **C)** Inhibitory concentration (IC) 90 values of the human-targeted drugs and the two macrolides erythromycin and azithromycin in mGAM on *B. uniformis* type strain and knockout strains. IC90 values are calculated based on the median normalized AUC of three biological replicates, with an AUC < 0.1 indicating the IC90. Heatmap shows the IC90 values in µM with a color code corresponding to the concentration range tested per drug. The label “>” means that even the highest concentration tested did not inhibit the strain by 90% or more (no IC90 reached).

To confirm that the loss of macrolide protection upon efflux pump inhibition is linked to the RND-type efflux pump operon (*BVU_1673–1675* in *P. vulgatus* and *BACUNI_00508–00510* in *B. uniformis*), we generated full operon knockouts: *P. vulgatus* Δ*BVU_1672–1675* (including the potential regulator *BVU_1672*) and *B. uniformis* Δ*BACUNI_00506–00510*. In both mutants, none of the three antagonists conferred cross-protection against erythromycin or azithromycin (Figures 4A and 5A).

In *P. vulgatus*, single-gene knockouts revealed that deletion of either the inner membrane protein (Δ*BVU_1674*) or the adapter protein (Δ*BVU_1673*) was sufficient to abolish dicumarol-mediated protection against both macrolides (Figure 4B). Deletion of the outer membrane pore protein (Δ*BVU_1675*) prevented erythromycin—but not azithromycin—protection, likely due to functional redundancy by another outer membrane protein, as seen with TolC in *E. coli.*^41^^,^^42^ Notably, deletion of the putative regulator (Δ*BVU_1672*) also abolished protection (Figure 4B).

*P. vulgatus* and *B. uniformis* encode multiple efflux pumps of various types (Supplementary Table 5). In *B. uniformis*, one ABC-type pump (BACUNI_01926–01932) is notably upregulated in response to dicumarol exposure (Figure 2D and Supplementary Table 4). Deletion of this ABC-type pump operon in *B. uniformis* (Δ*BACUNI_01926–01932*) had no impact on dicumarol-mediated macrolide protection (Figure 5B). Cross-protection was only abolished when this deletion was combined with the knockout of the RND-type efflux pump BACUNI_00508–00510. Similarly, the knockouts of additional efflux pumps in *P. vulgatus*: Δ*BVU_0379* (MATE-type), Δ*BVU_2346* (ABC-type inner membrane protein), Δ*BVU_2801* (ABC-type adapter protein), and Δ*BVU_3784* (RND-type outer membrane protein) retained the dicumarol-induced increase in macrolide IC90, similar to the wild type (Supplementary Figure 5A). This indicates that the cross-protection effect is specific to BVU_1673–1675 or BACUNI_00508–00510, respectively.

Importantly, none of the tested knockouts in either species significantly affected baseline IC90 values for macrolides or human-targeted drugs (Figures 4C, 5C and Supplementary Figure 5B), except for a modest increase in macrolide sensitivity in *P. vulgatus* Δ*BVU_1672–1675*, suggesting that these RND-type efflux pumps do not play a decisive role in macrolide resistance in the absence of the antagonists.

In summary, our findings demonstrate that the RND-type efflux pumps BVU_1673–1675 in *P. vulgatus* and BACUNI_00508–00510 in *B. uniformis* are essential for the protective effects of human-targeted drugs against macrolides.

### Dicumarol can induce and drive the evolution of increased macrolide resistance in *P. vulgatus*, dependent on the RND-type efflux pump BVU_1673–1675

Efflux pumps are known mediators of antibiotic resistance in bacteria,^43^^,^^44^ and their induction by human-targeted drugs may represent an unrecognized risk factor for the development of (multi)drug resistance. To determine whether human-targeted drugs need to be present during macrolide exposure to provide protection, we tested whether dicumarol can trigger or promote the development of increased macrolide resistance that remains even after dicumarol is removed.

**Figure 6.**
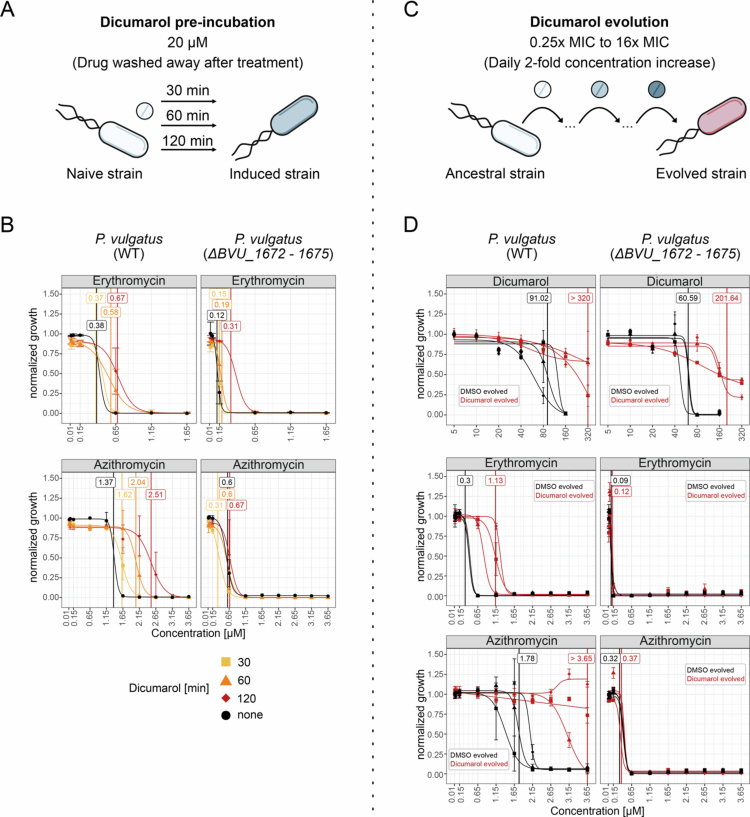
Protection from macrolides in *P. vulgatus* does not require the presence of the antagonists but can be induced and evolved with dicumarol. **A)** Schematic overview of dicumarol induction of *P. vulgatus*. Bacteria were adjusted to OD 0.02 and pre-induced in mGAM with 20 µM of dicumarol for 30 min, 60 min, or 120 min or left untreated. Cells were washed with PBS before measuring drug sensitivity. **B)** Dose response curves of untreated (black) and dicumarol-induced *P. vulgatus* after 30 min (yellow), 60 min (orange), or 120 min (red) when treated with different concentrations of either erythromycin (top) or azithromycin (bottom). Vertical lines indicate calculated inhibitory concentration (IC) 50 values. The wild-type is shown on the left and the Δ*BVU_1672−1675* mutant on the right. Three biological replicates per strain and condition. **C)** Schematic overview of *P. vulgatus* evolution with dicumarol. Bacteria were grown sequentially (1:600 dilution) in increasing concentrations of dicumarol in mGAM, starting at 0.25x inhibitory concentrations reducing 90% growth (IC90) (20 µM) to 16x IC90 (1,280 µM), with a two-fold concentration increase every 24 h. **D)** Dose response curves of DMSO evolved *P. vulgatus* (black) and dicumarol evolved lineages after growth in 16x IC90 dicumarol (red) when treated with different concentrations of either dicumarol (top), erythromycin (middle) or azithromycin (bottom). Vertical lines indicate calculated inhibitory concentration (IC) 50 values. The type strain is shown on the left and the Δ*BVU_1672−1675* mutant on the right. Three lineages per strain and condition were tested in three biological replicates.

To assess resistance induction, we pre-incubated the *P. vulgatus* wild-type and the Δ*BVU_1672–1675* knockout with 20 µM dicumarol for 30, 60, or 120 minutes before determining erythromycin and azithromycin IC50 values (Figure 6A). In the wild-type, erythromycin IC50 increased from 0.38 µM to 0.67 µM, and azithromycin IC50 from 1.37 µM to 2.51 µM, in a time-dependent manner (Figure 6B, left). In contrast, the knockout strain showed no IC50 shift, with only a modest growth increase at 0.15 µM erythromycin after 2 hours, and no change with azithromycin (Figure 6B, right). These results align with transcriptomic and proteomic data showing induction of BVU_1673–1675 within 30 minutes of dicumarol exposure (Figure 2).

Taken together, these findings show that a brief pre-treatment with dicumarol is sufficient to confer cross-protection against macrolides. As this effect persists in the absence of dicumarol, it does not necessitate the presence of the antagonist. Rather, sequential exposure is sufficient to confer cross-protection.

Since dicumarol can induce increased macrolide resistance, we hypothesized that continuous exposure could drive the evolution of *P. vulgatus* lineages with enhanced resistance to erythromycin and azithromycin. To test this, we performed an experimental evolution assay using the *P. vulgatus* wild-type and the Δ*BVU_1672–1675* knockout. Both strains were exposed to increasing concentrations of dicumarol (0.25 × to 16 × IC90), doubling every 24 hours (Figure 6C), while in parallel, control lines were exposed to solvent only (1% DMSO). Both strains evolved increased resistance to dicumarol (Figure 6D, top). However, only the wild-type evolved increased resistance to erythromycin (IC50: 1.13 µM vs. 0.3 µM in DMSO) and azithromycin (IC50: > 3.65 µM vs. 1.78 µM in DMSO), while the knockout showed no change (Figure 6D, middle and bottom). These results, consistent with the induction experiments, confirm that the RND-type efflux pump BVU_1672–1675 is essential for dicumarol-driven evolution of macrolide resistance, which persists without dicumarol.

## Discussion

Although there is growing recognition that non-antibiotic drugs can affect gut commensals both *in vitro*^24^^,^^45^^,^^46^ and in humans,^12^ the consequences of these interactions remain poorly understood. Here, we show that even brief exposure to certain human-targeted drugs—across distinct chemical and pharmacological classes and at sub-inhibitory concentrations— can trigger protection against clinically important macrolide antibiotics. Specifically, we demonstrate that certain non-antibiotic drugs induce the RND-type efflux pump BVU_1673−1675 in *P. vulgatus* and its homolog in *B. uniformis*. Importantly, these findings open new avenues for therapeutic strategies. The ability of non-antibiotic compounds to transiently and selectively induce efflux pumps in *Bacteroidaceae* suggests they could be used to protect commensal bacteria during antibiotic treatment.^14^ This may reduce collateral damage to beneficial microbiota, lower antibiotic-driven selective pressure, and enhance resistance to pathogen colonization. Notably, this induction can result in macrolide resistance phenotypes that persist even after washing out the drug. Thus, these antagonists must be used cautiously to avoid selecting for strains that constitutively upregulate efflux pumps, which would undermine their long-term applicability.

Recently, comparing chemical-genetic profiles from genome-wide knockout library screens has emerged as a powerful framework for systematically identifying cross-resistance between drugs. In *E. coli*, this approach has revealed numerous instances of both collateral sensitivity and cross-resistance among antibiotics, demonstrating its effectiveness for predicting drug-drug interactions.^47^ Extending such methods to gut commensals is crucial for understanding how a broader range of drugs influences antibiotic activity beyond classical pathogens and model organisms. Early efforts in this direction include transposon library screens in *Bacteroides thetaiotaomicron* and analyzes of drug–drug interactions across various bacterial species.^26^^,^^48^^,^^49^ Building on these foundations, our finding that certain compounds selectively induce RND-type efflux pumps suggests that large compound libraries could be systematically screened for efflux pump activation. This would facilitate the identification of antagonistic compounds for follow-up drug-drug interaction testing and help uncover the molecular features of antagonists that drive efflux induction. Ultimately, antagonists with no activity on the host could be used to protect the gut microbiome during macrolide treatment.

Our findings have important implications for understanding antimicrobial resistance in the complex environment of the human gut, where bacteria are exposed not only to antibiotics but also to a wide array of host-derived and pharmaceutical compounds. In pathogens, efflux pumps—particularly those of the RND family^50‐52^ —are known as key mediators of antibiotic resistance,^43^^,^^44^owing to their broad substrate specificity and ability to export macrolides.^52‐56^ These pumps can be induced by antibiotics,^57^^,^^58^biocides,^59^and environmental cues such as indole and bile,^43^^,^^44^^,^^58^and have also been shown to respond to non-antibiotic drugs.^60^^,^^61^ For instance, antidepressants have been reported to upregulate efflux pumps and increase antibiotic resistance in *E. coli.*^62^ Our data extend these observations to additional non-antibiotic compounds and reveal selective induction of specific efflux pumps within *Bacteroidaceae*. In our study, human-targeted drugs with minimal intrinsic antimicrobial activity triggered efflux pump expression, thereby enhancing resistance to an antibiotic that does not itself induce the pump. Importantly, deletion of the pump had little effect on the IC90 of either compound when tested separately, indicating that the inducing drug is likely not a pump substrate. This uncoupling of pump induction from substrate specificity exposes a latent resistance phenotype that remains hidden in standard susceptibility tests, which typically assess antibiotics in isolation. Importantly, such interactions make it difficult to predict resistance phenotypes based solely on (meta)genomic data.

From an evolutionary perspective, the efflux pump likely evolved to detoxify xenobiotics (structurally similar to the antagonists) rather than antibiotics, illustrating an example of exaptation where ancestral defense systems confer unintended antibiotic resistance in response to modern drugs.^63^ This broadens the evolutionary framework of antimicrobial resistance beyond direct antibiotic selection, supporting that resistance traits can emerge as collateral effects of bacterial adaptation to host and environmental chemical landscapes.^64^ This uncovers a previously underappreciated risk in microbiome–drug interactions: host medications may reshape bacterial resistance profiles not by selecting for resistance, but by transiently activating latent defense pathways.

Consequently, resistance traits in commensal *Bacteroidaceae* could complicate the treatment of anaerobic infections—such as intra-abdominal abscesses and post-surgical complications—and may position these microbes as silent reservoirs of resistance genes. In particular, the persistence of efflux-mediated resistance after the inducing compound is removed is concerning. Individuals exposed to such compounds may unknowingly harbor (transiently) resistant *Bacteroides* populations, potentially reducing the effectiveness of future antibiotic treatments or increasing the risk of difficult-to-treat infections. There are also broader epidemiological implications, as resistant microbes shed in feces could facilitate the spread of resistance genes in the environment. Moreover, although our three identified antagonists do not protect pathogens from macrolides,^14^ it remains important to assess whether these or other compounds can induce cross-resistance in enteric pathogens or alter their resistance dynamics.

In summary, our study shows that non-antibiotic pharmaceuticals can indirectly influence macrolide susceptibility by inducing efflux pumps. This highlights the complex interplay between host pharmacology and microbial responses in shaping antimicrobial susceptibility in complex environments. It also underscores the need to expand antimicrobial stewardship to account for microbiome-wide effects of all therapeutics—not just antibiotics, but also their combinations with other stressors.

## Supplementary Material

Supplementary Material Supplementary Tables

Supplementary Material Supplementary file

Supplementary Material Supplementary-Figures

## Data Availability

The mass spectrometry proteomics data have been deposited to the ProteomeXchange Consortium via the PRIDE partner repository with the dataset identifier PXD066285. The transcriptomics data generated in this study is available at the European Nucleotide Archive, accession ID PRJEB94528.
