# Supplementary material for: Antagonistic drug interactions protect commensal Bacteroidaceae from macrolides via an RND-type efflux pump
Source: Gut Microbes. 2025 Dec 9;17(1):2596806. doi: 10.1080/19490976.2025.2596806 (PMC12694919; doi:10.1080/19490976.2025.2596806)
Supplement: Supplementary Material — Supplementary file [file KGMI_A_2596806_SM2589.docx]

**Supplementary Figure 1**

**Checkerboard heatmaps of different *Bacteroidales* species against azithromycin.** Checkerboard analyses of different *Bacteroidales* grown in mGAM with increasing concentrations of the macrolide azithromycin in combination with increasing concentrations of the three human-targeted drugs dicumarol, benzbromarone and tolfenamic acid. Heatmaps show the median area under the growth curve (AUC) of three biological replicates normalized to the growth of the unperturbed control. Gray tiles indicate missing conditions that failed quality control.

**Supplementary Figure 2**

**The coumarin warfarin shows only weak antagonistic activity against azithromycin in *Bacteroidales* species. A)** Checkerboard analyses of *P. vulgatus* type strain grown in mGAM with increasing concentrations of the macrolide erythromycin or azithromycin in combination with increasing concentrations of warfarin. **B)** Checkerboard analyses of different *Bacteroidales* grown in mGAM with increasing concentrations of the macrolide azithromycin in combination with increasing concentrations of warfarin. **A) - B)** Heatmaps show the median area under the growth curve (AUC) of three biological replicates normalized to the growth of the unperturbed control.

**Supplementary Figure 3**

**Chemically similar coumarins do not protect against the macrolide erythromycin. A)** Tanimoto scores of chemical similarity for all investigated coumarins, warfarins and indandiones. The closer the value to 1, the higher the chemical similarity of two compounds. **B)** Checkerboard analyses of *P. vulgatus* type strain grown in mGAM with increasing concentrations of the macrolide erythromycin in combination with increasing concentrations of coumarins, warfarins or indandiones (in µM). Heatmaps show the median area under the growth curve (AUC) of three biological replicates normalized to the growth of the unperturbed control.

**Supplementary Figure 4**

**Oxygen and high salt stress do not protect against the macrolide erythromycin.** Checkerboard analyses of *P. vulgatus* type strain grown in mGAM with increasing concentrations of the macrolide erythromycin in combination with increasing concentrations of high salt stress with sodium chloride (NaCl) or oxygen in the form of hydrogen peroxide (H_2_O_2_). Heatmaps show the median area under the growth curve (AUC) of three biological replicates normalized to the growth of the unperturbed control.

**Supplementary Figure 5**

**Other efflux-pump gene knockouts do not affect protection from macrolides by antagonists in *P. vulgatus*. A)** Checkerboard analyses of single efflux-pump gene knockouts in *P. vulgatus* grown in mGAM with increasing concentrations of the macrolide erythromycin or azithromycin in combination with increasing concentrations of dicumarol. Heatmaps show the median area under the growth curve (AUC) of three biological replicates normalized to the growth of the unperturbed control. **B)** Inhibitory concentration (IC) 90 values of the human-targeted drugs and the two macrolides erythromycin and azithromycin on *P. vulgatus* type strain and knockout strains in mGAM. IC90 values are calculated based on the median normalized AUC of three biological replicates, with an AUC < 0.1 indicating the IC90. Heatmap shows the IC90 values in µM with a color code corresponding to the concentration range tested per drug. The label “>” means that even the highest concentration tested did not inhibit the strain by 90% or more (no IC90 reached).
