# Supplementary material for: Antagonistic drug interactions protect commensal Bacteroidaceae from macrolides via an RND-type efflux pump
Source: Gut Microbes. 2025 Dec 9;17(1):2596806. doi: 10.1080/19490976.2025.2596806 (PMC12694919; doi:10.1080/19490976.2025.2596806)
Supplement: Supplementary Material — Supplementary-Figures [file KGMI_A_2596806_SM2588.pdf]

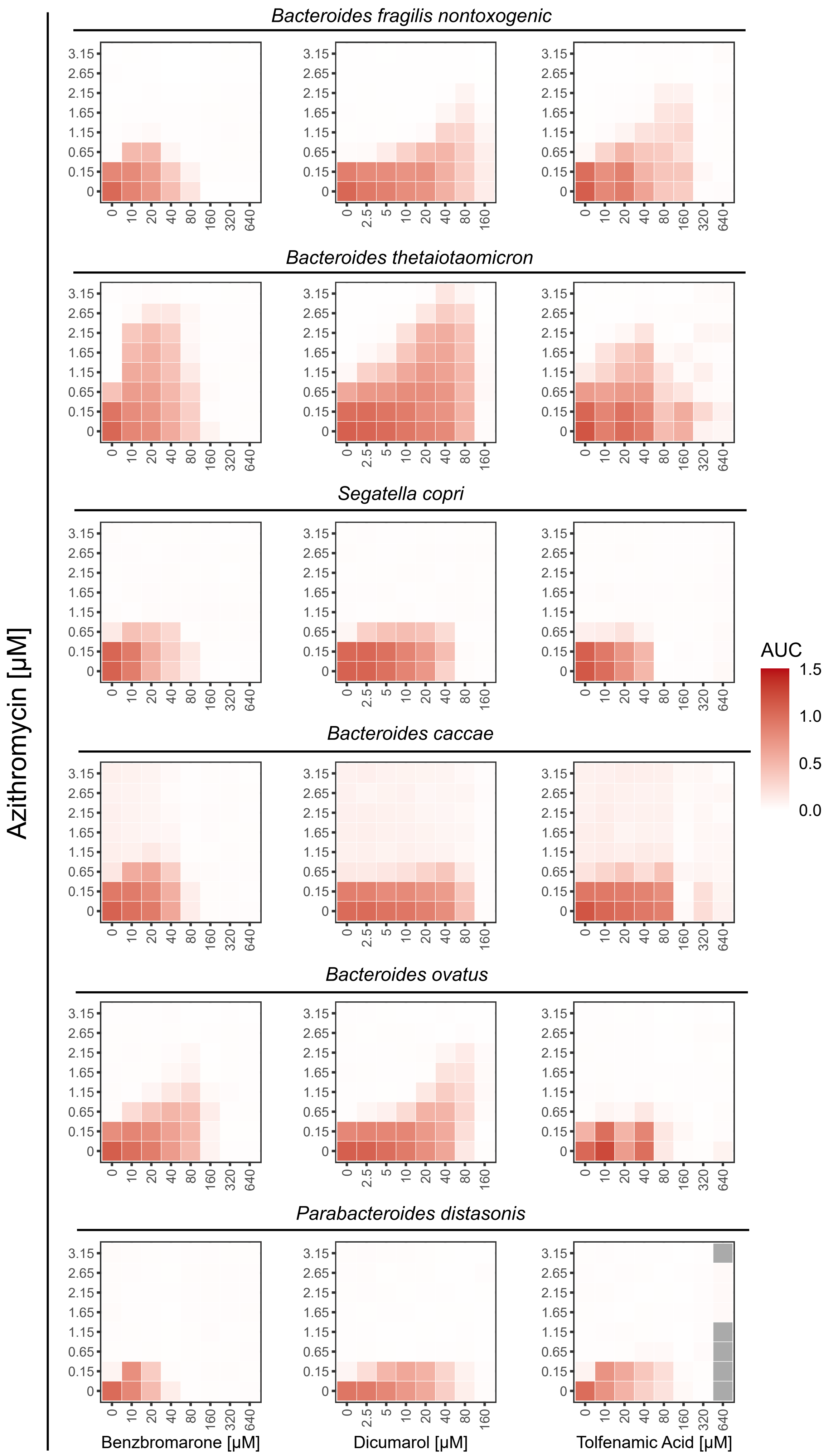

Supplementary Figure 1

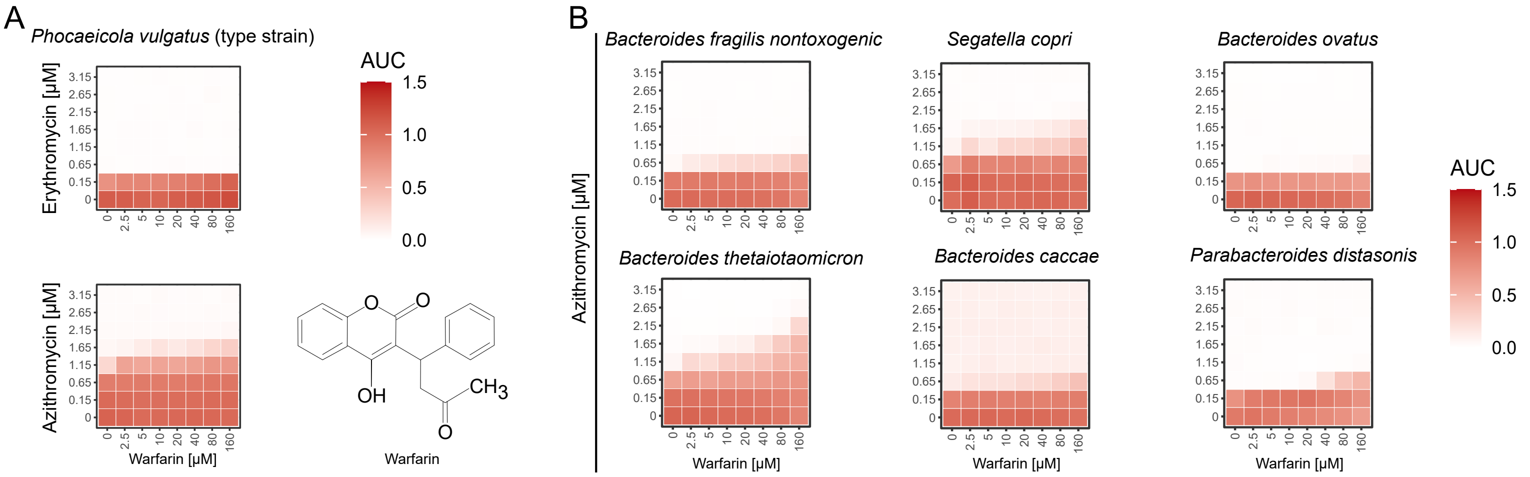

Supplementary Figure 2

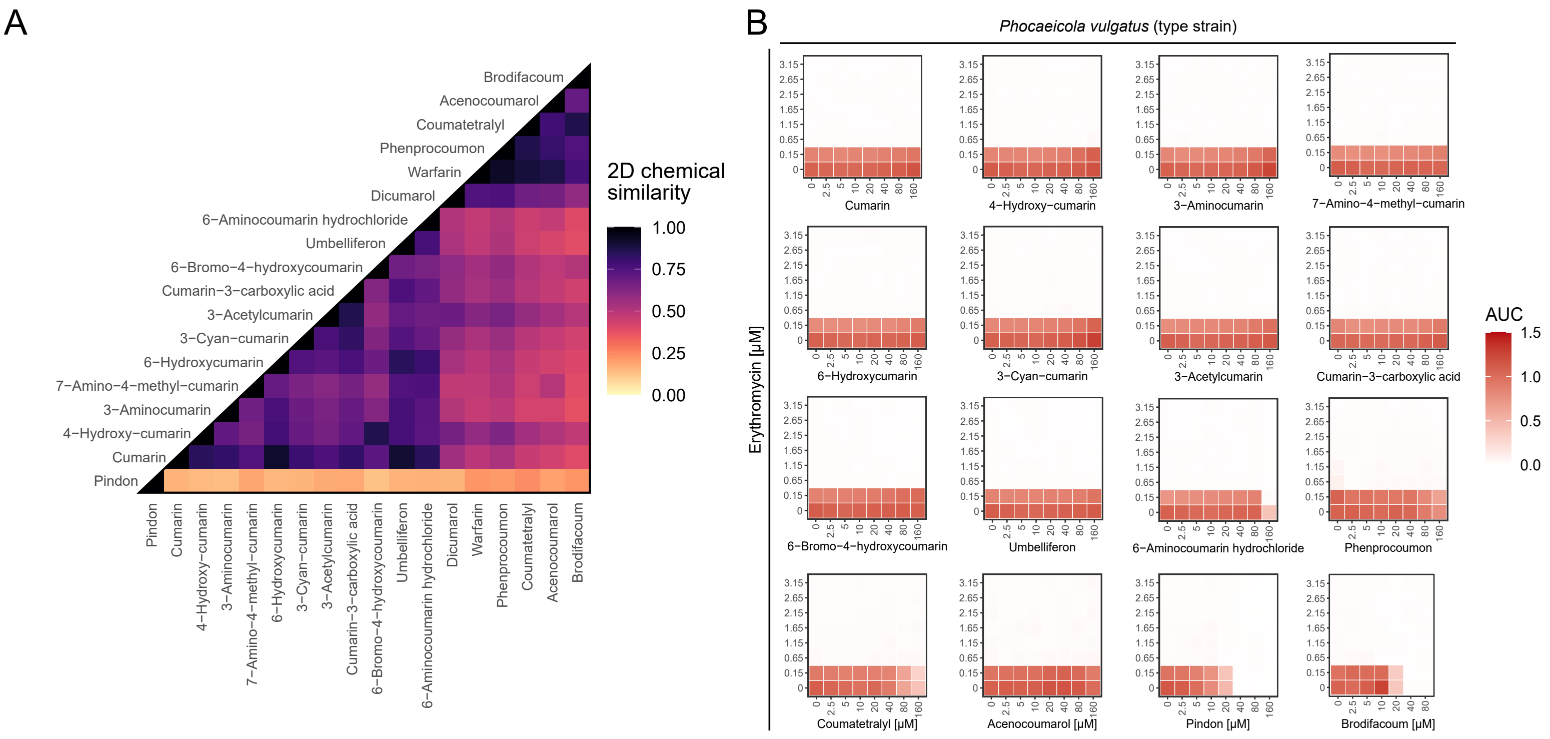

Supplementary Figure 3

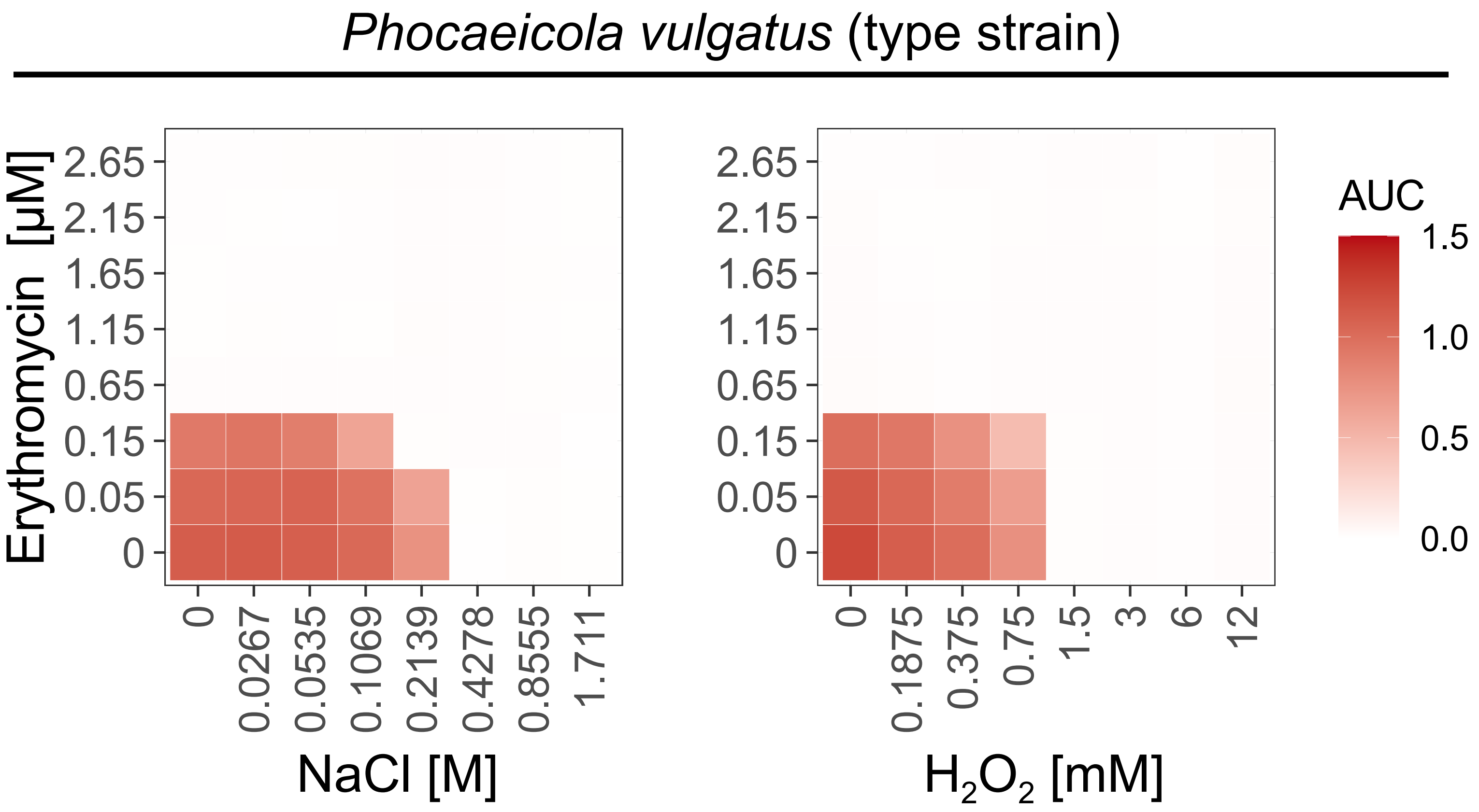

Supplementary Figure 4

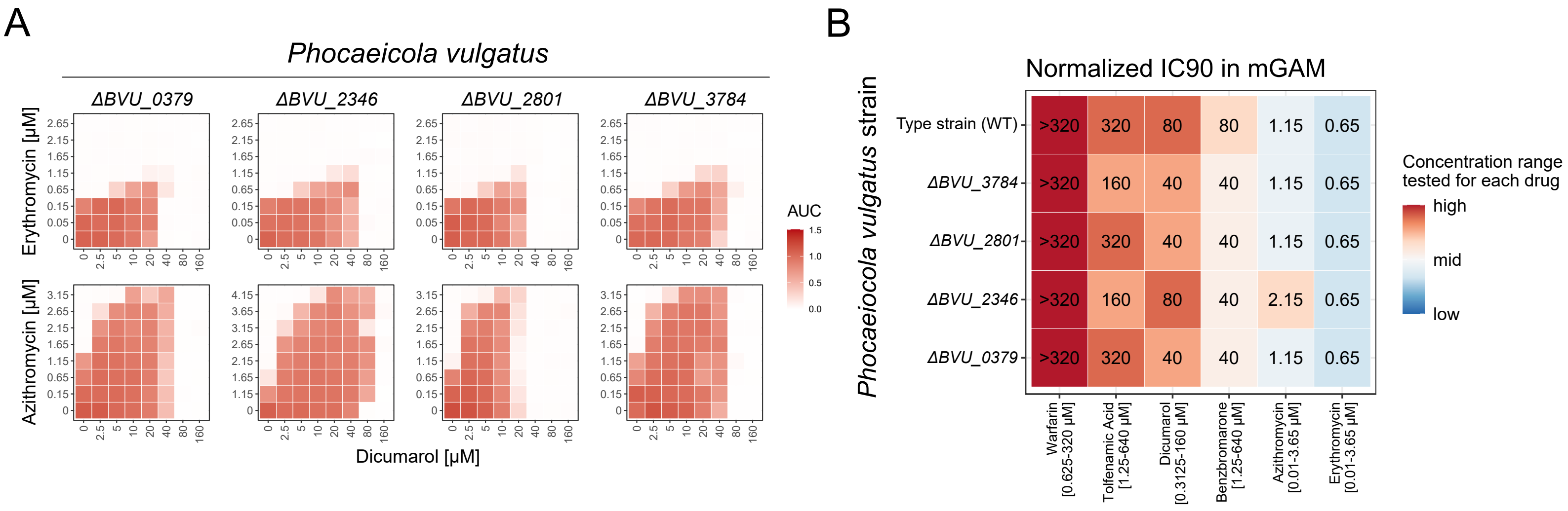

Supplementary Figure 5
